# Potential diabetic cardiomyopathy therapies targeting pyroptosis: A mini review

**DOI:** 10.3389/fcvm.2022.985020

**Published:** 2022-08-18

**Authors:** Yu Jia, Dongze Li, Jing Yu, Wenli Jiang, Xiaoyang Liao, Qian Zhao

**Affiliations:** ^1^General Practice Ward/International Medical Center Ward, General Practice Medical Center, West China Hospital, Sichuan University, Chengdu, China; ^2^Department of Emergency Medicine and National Clinical Research Center for Geriatrics, Disaster Medicine Center, West China Hospital, Sichuan University West China School of Medicine, Chengdu, China; ^3^Institute of Biomedical Engineering, West China School of Basic Medical Sciences and Forensic Medicine, Sichuan University, Chengdu, China

**Keywords:** diabetic cardiomyopathy, pyroptosis, NLRP3, caspase-1, pharmacology

## Abstract

Pyroptosis is primarily considered a pro-inflammatory class of caspase-1- and gasdermin D (GSDMD)-dependent programmed cell death. Inflammasome activation promotes the maturation and release of interleukin (IL)-1β and IL-18, cleavage of GSDMD, and development of pyroptosis. Recent studies have reported that NLRP3 inflammasome activation-mediated pyroptosis aggravates the formation and development of diabetes cardiomyopathy (DCM). These studies provide theoretical mechanisms for exploring a novel approach to treat DCM-associated cardiac dysfunction. Accordingly, this review aims to summarize studies that investigated possible DCM therapies targeting pyroptosis and elucidate the molecular mechanisms underlying NLRP3 inflammasome-mediated pyroptosis, and its potential association with the pathogenesis of DCM. This review may serve as a basis for the development of potential pharmacological agents as novel and effective treatments for managing and treating DCM.

## Introduction

Diabetes and heart failure have a bidirectional link. The prevalence of diabetes in patients with heart failure caused by cardiomyopathy ranges from 10 to 40% (1). Meanwhile, heart failure is a common and serious cardiovascular complication in patients with diabetes. The Framingham Heart Study showed that the incidence of heart failure is two- to fivefold higher in patients with diabetes compared with that in healthy individuals (2). In addition, pre-diabetes is also related to an elevated risk of heart failure, and the relative risks is 1.09–1.40 according to different diagnosis criteria (3). Notably, both pre-diabetes and diabetes are associated with an increasing risk of cardiac events and mortality in patients with heart failure (4, 5). Therefore, glycometabolism disorder is an important hazard factor for heart failure, and the two potential mechanisms are as follows: promoting the development of coronary atherosclerotic stenosis, which leads to ischemic heart disease characterized by systolic dysfunction; and more importantly, the classic presentation of diabetes, namely, diabetes cardiomyopathy (DCM) (1).

Diabetes cardiomyopathy is characterized by cardiac changes in function, metabolism, and structure without typical chronic cardiovascular complications, such as valvular heart disease, hypertension, and ischemic heart disease (6). DCM is the most frequent complication of diabetes and causes myocardial fibrosis, ventricular enlargement, and cardiac dysfunction, ultimately leading to clinical heart failure (7–9). Owing to its substantial impact on individuals cardiovascular health and lack of relevant targeted therapy, the pathogenesis of DCM has been a trending theme of research.

The abnormal metabolism of DCM is primarily due to myocardial tissue insulin resistance, compensatory hyperinsulinemia, and hyperglycemia, resulting in several conditions, including glycolipid metabolic disorders, oxidative stress, and advanced glycation end product deposition (1, 10). Previous review had well summarized the mechanisms of DCM, such as mitochondrial dysfunction, endoplasmic reticulum stress, and inflammation (11–13). Among the multiple mechanisms of DCM, cardiomyocyte death is a terminal pathway during the development of DCM, following by systolic dysfunction, myocardial compensatory hypertrophy, cardiac fibrosis, and electrocardiographic conduction disorder (14). Previous studies have analyzed that development of DCM caused by cardiomyocyte death, involving apoptosis, autophagy, necrosis, and entosis, and recent evidence obtained using electron microscopy has shown that pyroptosis-regulated cell death (pyroptosis) is a key pathogenetic factor in diabetes and DCM (15–17). Subsequently, an increasing number of pre-clinical studies have investigated the association between pyroptosis and DCM. Several molecular mechanisms have been elucidated, however, further related research is warranted.

## Mechanisms of pyroptosis

Pyroptosis presented as programmed and inflammatory cell death and characterized by caspase-1- and gasdermin D (GSDMD)-mediated formation of plasma membrane pores, following by cell lysis and the secretion of proinflammatory cytokines, such as IL-1β and IL-18, and cellular component (18). Pathogen associated molecular patterns (PAMPs) and damage associated molecular patterns (DAMPs) are identified by pattern recognition receptors (PRRs) to activate the intrinsic immune reaction (19–22). PRRs is divided into cell membrane PRR and cytoplasmic PRR according to receptors site. The former is expressed on the membrane of immunocyte, commonly known as Toll-like receptors (TLRs), which can identify the exogenous infection signals of the intracellular environment (20). The latter expressed in cytoplasm, it can identify invasive pathogens; The most common are retinoic acid-inducible gene I-like receptors, absent in melanoma 2 (AIM2)-like receptors (ALRs) and nucleotide-binding oligomerization domain (NOD)-like receptors (NLRs) (22–25).

**FIGURE 1:**
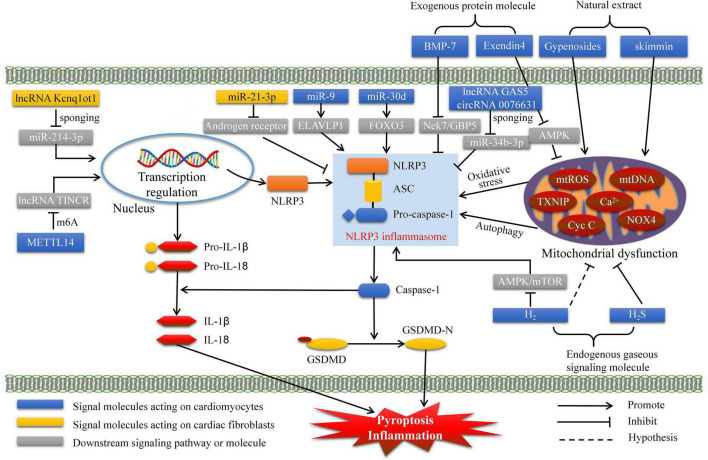
Pre-clinical treatment for DCM targeting NLRP3 inflammasome mediated pyroptosis. DCM, diabetes cardiomyopathy; GSDMD, gasdermin D; H2, hydrogen; H2S, hydrogen sulfide; METTL14, methyltransferase-like 14; BMP-7, bone morphogenetic protein-7; ELAVLP1, ELAV like protein 1; mtROS, mitochondrial reactive oxygen species.

When the ALRs and NLRs recognize DAMPs and PAMPs, the caspase-1 activated complex initiates assembly, this process is called formation of inflammasome (26). Further, it was regard as a processor of pro-caspases-1 to active caspase-1, which subsequently promoting maturation and release of IL-1β and IL-18 from precursor (26). Thus, inflammasome mainly contain three components: caspase-1, apoptosis-associated speck-like protein containing a caspase recruitment domain (ASC), and receptors. According to different receptors, inflammasomes mainly classified as AIM2, NOD-like receptor protein 1 (NLRP1), NLRP3, NLR family CARD-domain containing protein 4 (NLRC4), and NLRP6 inflammasomes. Although multiple kinds of inflammasomes are being intensively studied, NLRP3 inflammasome is currently the most studied, with the most abundant relevant evidence, and the most widely involved in inflammatory and immune diseases. Importantly, accumulating studies have revealed that NLRP3 inflammasome-mediated pyroptosis plays a significant role in inducing the formation and development of DCM. Thus, our current review aims to elucidate the molecular mechanisms of NLRP3 inflammasome-mediated pyroptosis and its potential association with the pathogenesis of DCM (Figure 1). Finally, we summarized the progress of clinical drug research for DCM targeting pyroptosis (Table 1).

**TABLE 1:** Clinical registered studies for diabetic cardiomyopathy targeting pyroptosis.

Registration time	Identifier	Phase	Study title	Conditions	Interventions	Status	Primary outcome measures
2012	NCT01752842	Unknown	Lipid Biomarkers for Diabetic Heart Disease	. Type 2 diabetes mellitus . Diabetes complications	Drug: fenofibrate Drug: placebo	Completed	Change in cardiac diastolic function as measured by E′ and fractional shortening percent
2019	NCT04200586	IV	The Effects of SGLTi on Diabetic Cardiomyopathy (SGLTi)	. Type 2 diabetes . Heart failure with reduced ejection fraction	Drug: dapagliflozin Drug: placebo	Active, not recruiting	Rate of change in myocardial T1 values with manganese enhanced cardiac MRI
2019	NCT01803828	IV	REmodelling in Diabetic CardiOmyopathy: Gender Response to PDE5i InhibiTOrs (RECOGITO)	. Diabetic cardiomyopathy . Diabetes mellitus type 2	Drug: tadalafil Drug: placebo	Completed	Change from baseline in left ventricular torsion
2019	NCT04141475	Unknown	Evaluation of Alpha-Lipoic Acid in Diabetic Cardiomyopathy (CARDIALA)	. Diabetic cardiomyopathies	Drug: physiomance acide lipoïque gold Drug: placebo	Recruiting	Change of LVEF between before and after 12 weeks of treatment
2020	NCT04591639	IV	The DAPA-MEMRI Trial (DAPA-MEMRI)	. Heart failure . Diabetic cardiomyopathies	Drug: dapagliflozin Drug: placebo	Recruiting	Change in myocardial perfusion reserve index
2022	NCT04083339	III	Safety and Efficacy of AT-001 in Patients With Diabetic Cardiomyopathy	. Diabetic cardiomyopathy	Drug: AT-001 Drug: placebo	Recruiting	Peak VO2 during cardio-pulmonary exercise test

## Pre-clinical diabetes cardiomyopathy treatments targeting nucleotide-binding oligomerization domain-like receptor protein 3 inflammasome-mediated pyroptosis

### Natural extracts

Cardiac pumping dysfunction in DCM is mainly due to cardiomyocyte injury and death. Thus, suppressing pyroptosis in cardiomyocytes is important. Gypenosides, the principal component of *Gynostemma*, exert various cardiovascular protective effects, such as reducing blood pressure, improving lipid and glucose metabolism, and inhibiting inflammation (27, 28). Zhang et al. reported that gypenosides can ameliorate high glucose-induced DCM by inhibiting reactive oxygen species (ROS)- and cytochrome c-mediated NLRP3 inflammasome activation and pyroptosis (29). Skimmin is a coumarin and glycoside with several biological activities, including antifibrosis, antioxidation, and anti-inflammation (30, 31). A recent study has shown that skimmin protects against streptozotocin (STZ)-induced DCM by improving autophagy and inhibiting NLRP3 inflammasome-mediated pyroptosis in rat cardiac tissues. Therefore, ginsenosides and skimmin are promising therapeutic drugs for DCM treatment.

### Non-coding RNAs

Studies in recent years have recognized the important physiological roles of non-coding RNAs (ncRNAs), including circular RNAs (circRNAs), long ncRNAs (lncRNAs), and microRNAs (miRNAs) (32). These ncRNAs are related to DCM through transcriptional and post-transcriptional regulation but not directly involved in protein translation. For example, miRNA-9 expression is downregulated in the cardiac tissue of patients with diabetes and in cardiomyocytes treated with high glucose (33). Furthermore, miRNA-9 mimics inhibit pyroptosis (determined from the caspase-1 and IL-1β levels) in cardiomyocytes by targeting ELAV-like protein 1 to ameliorate hyperglycemia-induced DCM (33). Moreover, Li et al. observed that miR-30d level was increased and in STZ-treated diabetic rat hearts and high glucose-induced cardiac cell. Next, miR-30d was proved to inhibit Forkhead box O3 activities (apoptosis inhibitor) and exacerbates pyroptosis in DCM (34).

Xu et al. confirmed that GAS5 sponges miR-34b-3p to promote aryl hydrocarbon receptor expression and subsequently suppresses NLRP3 inflammasome-mediated pyroptosis in cardiomyocytes to alleviate DCM (35). Interestingly, Yang et al. reported that caspase-1-associated circRNA (hsa_circ_0076631) also sponges miR-214-3p (endogenous) to enhance high glucose-treated NLRP3 inflammasome activation and pyroptosis in cardiomyocytes (36). In addition, epigenetic regulation of lncRNAs can be modified by N6-methyladenosine (m6A), whose level and activity impact cell pathophysiology (37). Recent research has reported that the overexpression of the lncRNA TINCR enhances cardiomyocyte NLRP3 inflammasome activities, pyroptosis, and DCM, and the epigenetic regulation of TINCR is controlled by methyltransferase-like 14-mediated m6A methylation.

In addition to cardiomyocytes, cardiac fibroblasts are also vulnerable to high glucose levels and exacerbate fibrosis and DCM. Yang et al. reported that lncRNA Kcnq1ot1 activates the caspase-1 and TGF-β1 pathways to aggravate fibrosis and DCM by sponging miR-214-3p in cardiac fibroblasts. Moreover, miR-21-3p expression in cardiac fibroblasts is upregulated under STZ treatment, whereas functional inhibition of miR-21-3p improves pyroptosis and collagen deposition by elevating the androgen receptor. These studies demonstrate that ncRNAs play crucial roles in DCM pathogenesis.

### Endogenous gaseous signaling molecules

Endogenous gas signaling molecules serve important physiological and pharmacological functions and are associated with diabetes and related complications. Kar et al. found that hydrogen sulfide can be regulated by physical exercise and serves as a cardioprotective antioxidant that suppresses the activation of NLRP3, IL-1β, IL-18, and caspase-1 (38). Another recent study has demonstrated that hydrogen inhibits cardiomyocyte pyroptosis in cardiac fibroblasts by blocking the AMPK/mTOR/NLRP3 signaling pathway and improves fibrosis by inhibiting the TGF-β1/Smad signaling pathway (39). Moreover, hydrogen, as a therapeutic antioxidant, can reduce intracellular oxygen free radicals and inhibit ROS production (40). Thus, hydrogen inhibits the pathogenesis of DCM through multiple pathways. Hydrogen sulfide and hydrogen have been validated as gaseous signaling molecules that prevent DCM by alleviating pyroptosis.

### Exogenous protein molecules

Bone morphogenetic protein-7 (BMP-7), also known as osteogenic protein-1, is used in clinical medicine to treat osteoporosis and fracture (41). BMP-7 inhibits inflammation and improves neovascularization (42, 43). Furthermore, BMP-7 inhibits NLRP3 inflammasome-mediated pyroptosis by blocking Nek7/GBP5 signaling to improve deleterious cardiac function and remodeling (44). Exendin-4, a glucagon-like peptide-1 analog, has an extended half-life because it avoids the clearance of dipeptidyl peptidase IV (45). Numerous studies have emphasized its protective effects on glucose metabolism and cardiac function (46). Additionally, exendin-4 inhibits pyroptosis *via* the ROS/AMPK/TXNIP/NLPR3 pathway, indicating that exendin-4 is a potential therapeutic drug for DCM (47). Secreted frizzled-related proteins (SFRPs) are a family of secreted proteins, and they were characterized by negative regulation of pyroptosis through Wnt/β-catenin and Notch signaling pathways in cardiovascular disease and inflammatory disease (48, 49). Recent study demonstrated that SFRP5 is a powerful prognostic assessment factor of heart failure for patients with type 2 diabetes (T2D). Thus, SFRPs may be a novel and potential exogenous inhibitory molecules of DCM by targeting pyroptosis (50). It is innovative and significant to carry out related research.

## Clinical diabetes cardiomyopathy therapies targeting pyroptosis

### Sodium-glucose cotransporter-2 inhibitors

Sodium-glucose cotransporter-2 (SGLT2) inhibitors are a relatively new type of hypoglycemic drug that increases urinary glucose excretion for the treatment of T2D (51, 52). Several clinical trials have revealed that SGLT2 inhibitors exert powerful cardiovascular protective effects, such as empagliflozin, canagliflozin, and dapagliflozin, on patients with T2D (53–55). Furthermore, the EMPRISE trial verified that empagliflozin decreases the risk of hospitalization for heart failure in patients with T2D (56). However, these cardiovascular protective effects of SGLT2 inhibitors cannot be attributed merely to their hypoglycemic and natriuretic effects. Pre-clinical studies revealed that SGLT2 inhibitors attenuate myocardial oxidative stress, fibrosis, and DCM by inhibiting NLRP3 inflammasome-mediated pyroptosis in diabetic mouse heart (57–59). To date, clinical studies confirming that SGLT2 inhibitors can improve DCM are lacking, although two related clinical trials are in progress. The DAPA-MEMRI trial (Identifier: NCT04591639) enrolled heart failure patients with T2D from October 2020 to explore the protective effects of SGLT2 inhibitors on cardiac function and remodeling by using cardiac magnetic resonance imaging (MRI) and echocardiography (Table 1). The results of this study provide direct evidence that SGLT2 inhibitors can improve DCM in humans. However, the other study (Identifier: NCT04200586) has not recruited participants yet (Table 1). These results suggest that SGLT2 is a promising drug for DCM treatment.

### Phosphodiesterase type 5 inhibitors

Cyclic guanosine monophosphate-phosphodiesterase type 5 (PDE5) inhibitors have gained attention because they can alleviate cardiac stress responses and improve hypertrophy and cardiac damage from multiple adverse stimuli in clinical and pre-clinical studies (60, 61). Early studies in men with heart failure and preserved ejection fraction showed that PDE5 inhibitors improved pulmonary pressure, cardiac geometry, and pump function (62). A recent RECOGITO study (Identifier: NCT01803828) has enrolled 122 men and women with well-controlled T2D and revealed that treatment with 20 mg tadalafil for 20 weeks can significantly mitigate DCM in men but not in women (63) (Table 1).

Mechanistically, PDE5 inhibitors exert protective effects on the cardiovascular system by activating protein kinase G (PKG), PKG-dependent hydrogen sulfide generation, nitric oxide expression, and glycogen synthase kinase-3β phosphorylation (64–66). In addition to hydrogen sulfide-mediated NLRP3 activation (38, 39), the PDE-5 inhibitor TPN171H, an icariin derivative, displays significant anti-inflammatory activities *via* suppressing NLRP3 inflammasome-mediated pyroptosis *via* cathepsin B (67–69). These results indicate that PDE5 inhibitors provide cardioprotection against DCM by inhibiting NLRP3 inflammasome-mediated pyroptosis; however, direct evidence is still lacking.

### Aldose reductase inhibitors

Aldose reductase, as a polyol pathway enzyme, is significant upregulated in the conditions of oxidative stress and is the important inducer of the ROS related inflammatory response in diabetes (70). Pal et al. demonstrated that aldose reductase inhibitors prevent NLRP3 inflammasome-mediated pyroptosis and cytokine release in monocytes and STZ-induced diabetic mouse heart (71). Thus, aldose reductase inhibitors targeting NLRP3 inflammasome-mediated pyroptosis may be potential agents for DCM treatment. To the best of our knowledge, only one phase III trial (Identifier: NCT04083339) has been conducted to test the safety and efficacy of AT-001 (aldose reductase inhibitor) in patients with DCM. Although this study was started on 10 September 2019, the anticipated results have not been published yet (Table 1).

### Fenofibrate

Fenofibrate is a peroxisome proliferator-activated receptor α agonist that has been widely used in the clinic for several decades because of its remarkable effect of reducing triglycerides (72). It can ameliorate diabetic retinopathy and stimulate angiogenesis by deregulating the activity of the NLRP3 inflammasome in STZ-induced diabetic mice (73, 74). Fenofibrate exerts a considerable protective effect on the heart, but whether it can ameliorate DCM remains unclear. A randomized controlled study (Identifier: NCT01752842) tested whether 160 mg fenofibrate per day for 12 weeks can improve heart muscle function in patients with T2D (Table 1). However, results of this study revealed no significant difference in cardiac diastolic function as measured by E′ (cm/s) and fractional shortening percentage between the placebo and fenofibrate groups.

### Alpha-lipoic acid

Alpha-lipoic acid (ALA), also known as thioctic acid, is a vitamin-like sulfur-containing organic compound abundant in human organs and tissues (75). Early studies demonstrated that ALA is involved in improving hyperglycemia and deregulating inflammation (76–78). Recent studies have reported that ALA alleviates dyslipidemia and inflammation by modulating NLRP3 inflammasome activation in rats with high-fat diet- and STZ-induced T2D (79, 80). A randomized controlled study (Identifier: NCT04141475) involving patients diagnosed with diabetes from October 2019 evaluated the effect of ALA (Physiomance Acide Lipoïque Gold) in DCM by measuring the left ventricular ejection fraction (Table 1). The results of this study are worth investigating further.

## Conclusion and perspectives

Pyroptosis is primarily considered a pro-inflammatory class of caspase-1- and GSDMD-dependent programmed cell death *via* the NLRP3 inflammasome. An increasing number of preclinical studies have emphasized that pyroptosis, which is different from apoptosis and necrosis, is involved in the pathogenesis of DCM. For example, natural extracts (derivatives), ncRNAs, endogenous gaseous molecules, and exogenous proteins have been explored and recognized for their key roles in pyroptosis and DCM. These studies offer theoretical mechanisms for developing new drugs to treat DCM-related cardiac dysfunction in the future. In addition, some clinical studies are actively exploring marketed drugs that may treat DCM, such as SGLT2 inhibitors, PDE5 inhibitors, aldose reductase inhibitors, fenofibrate, and ALA. The pharmacology of these drugs involves the inhibition of NLRP3 inflammasome-mediated pyroptosis. Thus, they may be the earliest evidence-based medicine for clinical use. However, basic and clinical investigations are still warranted to establish novel and effective treatments targeting pyroptosis for managing and treating DCM.

## Author contributions

YJ, DL, and QZ designed the research. YJ and DL wrote the first draft of the manuscript. JY, QZ, WJ, and XL reviewed the manuscript and provided critical scientific input. QZ had main responsibility for the final content of the manuscript. All authors approved the final draft of the manuscript.
